# Acknowledgement to Reviewers of *Vaccines* in 2019

**DOI:** 10.3390/vaccines8010030

**Published:** 2020-01-16

**Authors:** Vaccines Editorial Office

**Affiliations:** MDPI, St. Alban-Anlage 66, 4052 Basel, Switzerland; vaccines@mdpi.com

The editorial team greatly appreciates the reviewers who have dedicated their considerable time and expertise to the journal’s rigorous editorial process over the past 12 months, regardless of whether the papers are finally published or not. In 2019, a total of 219 papers were published in the journal, with a median time to first decision of 21.27 days and a median time from submission to publication of 5.65 days. The editors would like to express their sincere gratitude to the following reviewers for their generous contribution in 2019:
Abbie FrancisAbishek ChandrashekarAbram WagnerAdam Wheatley Adriaan D. BinsAfsar RahbarAgathe ColmantAgnieszka AdamekAhmed MesalamAhmed MostafaAiqiang XuAlan CrouchAlberto A. AmarillaAlberto LaddomadaAlejandro BrunAlejandro Marin-LopezAlejandro Samhan-AriasAleksandra PawlakAlexander DomnichAlexandra SpencerAlfonso Martin-FontechaAlistair McGregorAllison C. VilanderAlok RanjanÁlvaro Francisco Lopes De SousaAlyson KelvinAmila GunesekeraAmy BaxterAna ReisAnders FomsgaardAnders HjernAndrea CaraAndrea KroekerAndrea KuemmerleAndres Jimenez Galisteo Jr.Andreu Comas-GarciaAndrew GetahunAndris DišlersAndy SuterAneesh ThakurAngela L. RasmussenAngela ArcielloAngela SousaAngelo IzzoAnisha Mahendra ThankiAnnalisa Del PreteAntonio Q. TonioloAntoniou AntonyAntonius (Tom) OomensAnwar Anwar MohamedArnaud MachelartAsuka NanboAtsushi YamanakaAudray K. HarrisAxel LehrerBaoliang PanBasem BattahBeatrice GolombBenjamin AnsaBenoit CallendretBert SchepensBill WangBinod KumarBo LiangBoris A. YakobsonBram SlütterBrian D. PooleBrian Patrick McSharryBrian R. WasikBruce A. RosaBruno BeaumelleBryce ChackerianCamila CoelhoCelia Alpuche-ArandaChad RoyChanin NantasenamatChao ShanCharlene KahlerCheng-Hsun ChiuChengzhong YuChithra SreenivasanChiu-Ming WenChristine E. EngelandChristine LuttermannChristopher MacRaildChristopher StobartChukwunonso NzeluChulhwan ParkChun Shen LimChung-Jung LiuClaudio CostantinoClement MesedaCristian SmerdouCyrille MathieuDagmara Weronika WójkowskaDaniel P. PotaczekDaniel Xin ZHANGDaniel DoryDaniel MarcDaniel O’ConnorDaniel SteinDaniela HozborDanny AltmannDarren J. WightDavid A. SchwartzDavid E. BrilesDavid Scott McVeyDavid MarchantDavid PejoskiDavide AngelettiDawn WeirDeb FullerDeborah FullerDeborah SpectorDenis LeclercDennis W. MetzgerDennis BenteDer-Shan SunDilshani SarathchandraDolores R. SerranoDomenico MattoscioDomenico TortorellaDonatella NegriDr. Edson Roberto Da SilvaDuo ZhangDylan M. JohnsonEd RybickiEdward MocarskiEdward StephensElizabeth RiederEllen CollissonEl-Sayed Abdel-WhabElvia Janeth OsorioEmilia HadziyannisEmilien JeannotEric HarvillEric WeaverEric YagerEskild PetersenEsther E. Biswas-FissEugene RyabovEva AndreuzziEwa BalcerczakFabrizio BertelloniFabrizio BruschiFabrizio VitaleFan ZhouFayna Diaz-San SegundoFederica FaciottiFelix StahlFeng GuanFernando FerreiraFernando SpilkiFiona EcarnotFrancesca AndreoniFrancesca FerraraFrancesca GattoFrancesca GilliFrancesca ManciantiFrancesca VasileFrancesco BonfanteFranklin D. EvansFrederick BaliraineFrieder KellerFriederike JönssonFun-In WangGabriel I. NistorGabriele A. LandoltGabriele MulthoffGavin PainterGaya AmarasingheGayle DeLongGenevieve André-FontaineGeoffrey LynnGeorg F. WeberGeorge B KyeiGeorge RachiotisGeorgina BowyerGer RijkersGer Van ZandbergenGertrud StegerGianluca BaldanziGillian L BeamerGiovanna RomeoGiovanni Battista Levi SandriGisselle N. MedinaGiuseppe A. SauttoGiuseppe Di MartinoGiuseppina BasiniGokhlesh KumarGopas JacobGrace M. CentolaGraham PawelecGraham WilliamsGregory KaradjianGuido VanhamGunjan AroraGunnveig GrodelandGus KousoulasHang XieHao ChengHeather CostelloHeather Stout-DelgadoHedwin Kitdorlang DkharHeidi J LarsonHeinzpeter SchwermerHenk-Jan SchuurmanHenk-Jan SchuurmanHenri-Jacques DelecluseHeshan Sam ZhouHiep VuHimanshu GargHinh LyHiraku SasakiHo Seong SeoHoang-Thanh LeHong XinHua ZhuHui-Chen ChenHui-Wen ChenI-Cheng Mark ChenIgnacio MenaIlaria ManiniIlkka JulkunenIrina V. KiselevaIrit DavidsonIsidoro MartínezIvan KuzminIvan Sanz MuñozJ. D. AlbarnazJack D. SunterJacob S. YountJames B. FinkJames BinleyJames CollinsJan KopeckyJaneen Salak-JohnsonJanice HegewaldJason E. ComerJason KaelberJason KindrachukJean-Pierre FrossardJefferson SantosJenna E. AchenbachJennifer KrissJeremy KamilJeroen CorverJerry M. TroutmanJerry W. SimeckaJessica SmithJiafen HuJillian FilliterJin-Sheng HeJinsong HaoJinyang ZhangJoanna DepciuchJoão Manuel Graça FradeJoão MesquitaJoão MesquitaJoe MymrykJoel PalefskyJohanne PoudrierJohn C. HaydenJohn T. BatesJohn AlcornJolanta RzymowskaJoon Haeng RheeJorge Galindo-VillegasJorge Gutierrez-MerinoJorgen De JongeJorma HinkulaJose Joaquin MerinoJosé Caparros-MartinJosé TuellsJoseph H. BanoubJoshua KennedyJuan B De SanctisJuan BarcenaJuan ZapataJudith AlferinkJuergen Schneider-SchauliesJulia DinaJun InoueJun MiyazakiJunji XingJussi HepojokiK. MegyeriKa-chun ChongKai HuangKarim VermaelenKate SeibKaterina ChlichliaKatherine SpindlerKathleen Laura HefferonKazunori NagasakaKazunori NagasakaKelly L. GorresKendra AlfsonKerry LavenderKevin D. PavelkoKevin HollevoetKevin McHughKevin PollockKinjiro MorimotoKiryl PiatkevichKobie James J.Kohei MakitaKonrad PisarskiKorbinian BrandKrzysztof TrzcińskiKumaraswamy Naidu ChitralaLai AlexanderLai AlexanderLalita PriyamvadaLarance RonsardLarry AndersonLaura GibsonLauren A. SiltzLauren O’DonnellLaurent Chatel-ChaixLaurent L’hommeLeonie Marloes VogtLeslie MeynLeyi WangLianne TrippLin WangLindsey CrawfordLisa K. RyanLogan BanadygaLoredana NicolettiLorna LealLouise CosbyLu ZhangLuka Cicin-SainLukas SchwarzLukasz KurykMa LuoMagdalena SzaryńskaMakoto TakedaMaksymilian GajdaMalaya Kumar SahooMarc GirardMarcelo SilvaMarcin W. BańburaMarco PittauMargaret LiuMargarita SáizMaria Cristina CaroleoMaria Grazia CusiMaría José AlcarazMaria T SanchezMaria BottazziMaria GrandahlMaria TempestaMariana BazMarie De PerioMarie-Hélène MayrandMarino PrearoMark Y. SangsterMark AldersonMark PeeplesMark SkidmoreMarta L. DeDiegoMartha A. Alexander-MillerMartin RafteryMartina JonesMartina ScallanMarvin GrubmanMary Patricia NowalkMarzena KamienicznaMasfique MehediMatthew MillerMatthew ReevesMauro PistelloMaxime PichonMeaghan HancockMehmet OzenMeik KunzMei-Ling LiMelinda BrindleyMelodie Yunju SongMichael A. BarryMichael A. SchotsaertMichael C BurgerMichael KoldehoffMichael McVoyMichael MinaMichael MuehlebachMichael WorobeyMichał GondekMichal PyzikMichele GhidiniMiguel A. Martín-AcebesMikolaj AdamekMilena KordalewskaMingyuan HanMirella SalvatoreMirko TrillingMohan Kumar Muthu KaruppanMohan AmarasiriMona O. MohsenMudit TyagiMunegowda KoralurNagarjuna Reddy CheemarlaNaoki InoueNaphak ModhiranNatalia SavelyevaNatalia SavelyevaNatasha N GaudreaultNeda BarjestehNereida Jiménez De OyaNicholas JohnsonNicholas JohnsonNicla RomanoNiels A W LemmermannNigel TempertonNikki TurnerNimish PatelNorbert PardiNorbert PardiNúria TornerNuruddin UnchwaniwalaOlivier SchildgenOrlando Torres-FernándezPablo CaballeroPablo CaballeroPablo Guardado-CalvoPamela K. StrohfusPamela WearschPanayampalli Subbian SatheshkumarParminder J. S. VigPartha KarmakarPatricia DevauxPaul BollykyPaul JohnsonPaul LicciardiPaul SpearmanPaul ZajacPaulo Lee HoPedro J. AlcoleaPedro FolegattiPeng ZhangPeter MaplePeter PalesePetr O. IlyinskiiPhillip GouldPiero LovreglioPierre BussonPiotr ZukPiyapong JanmaimoolPooja TiwariPooja TiwariPramod K. SrivastavaPrzemyslaw CwynarPullagurala Venkata Laxma ReddyPumtiwitt McCarthyQian WangQinjian ZhaoQinxue HuQiyi TangRafael C. Prados-RosalesRaffael NachbagauerRamon ArensRan XieRandy AlbrechtRavi S MisraRedmond SmythReed F. JohnsonReinhard ZelenyRemi CreusotRicardo Pérez-SánchezRich BennettRichard J.P. BrownRichard BowenRichard MarkhamRichard StantonRikhav P GalaRino RappuoliRita Cláudia Cardoso RibeiroRobert BransfieldRobert CharvatRobert CrossRoberto NisiniRobin P. Goin-KochelRohit JangraRoland ZüstRon GellerRonald M. IorioRory D. De VriesRussell KabirSABARINATH NEERUKONDASadik A. KhuderSailen BarikSailen BarikSara MangsboSarah B. ManessSashi KantSatabdi ChatterjeeSayantan BoseScott S. TerhuneScott W. MuellerScott FillerSebastian Maurer-StrohSebastian UlbertSekine ShinichiSeonju YeoSergei BazhanSergio Daishi SasakiSéverine PéchinéShailendra B. TallapakaShakti SinghSharan BobbalaShauna KaplanSheryl Van NunenShigetaka ShimodairaShigetaka ShimodairaShi-hua XiangShih-Yen LoShikha TarangShiro MurataShoujun LiShusaku MizukamiSinem BeyhanSJ HewlingsSonali BhattacharjeeSoon Young PaikSophie A. ValkenburgSouleymane DoucoureSpiridon MantzoukasSreeKumar BalanSri Ram PentakotaStefan NierkensStefania Mirela MangStefano MenzoStephane LemiereSteven BradfuteSteven RockmanSteven SylvesterSuh-Chin WuSupreet KhanalSveinung Wergeland SorbyeSven-Erik BehrensSvenn-Erik MamelundSwapnil BawageSwastik PhuleraTahir KhanTaisen IguchiTakayuki HoshinaTakeyuki ShimizuTamara GulicTaro KamigakiTatiana PintoTeiji SawaTerry Ann ElseTetsuo NakayamaTetsuro IkegamiThomas GrunwaldTian (Tina) WangTian DebinTimothy KurttiTingting ChenTodd G. SmithTomas HankeTomer HertzTomohiro IshikawaToshi NagataTsung-Hsien ChangUdo JeschkeUjjwal NeogiUrsula WiedermannVagner Ricardo LungeValentina A FeodorovaValéria Marçal Felix De LimaValerie MorleyVeronica JarockiVeronika DillVicky Van SantenVictor HuberVillyen MotazeVirginie RozotVitaly GanusovVivienne TippettWalid Mahmoud KhaliliaWanbo TaiWegene T. BorenaWeiguang ZengWeili KongWendy MauryWildriss ViranaickenWilliam JamesWon Hyung ChoiXavier SaelensXiaona YouXiaowu ChenXiaoying LiuXihong ZhaoXinxing ZhangXuefei HuangXueling WuYa-Fang MeiYang ZhaoYashar SadighYijun ZhangYin LiYi-Ning ChenYi-Yuan YangYong GaoYongjun SuiYongtang ZhengYoshihiko HirohashiYoung LeeYousuf MohammedZhicheng DouZhihong YuanZhilong Yang

